# Design of Flexible Hardware Accelerators for Image Convolutions and Transposed Convolutions

**DOI:** 10.3390/jimaging7100210

**Published:** 2021-10-12

**Authors:** Cristian Sestito, Fanny Spagnolo, Stefania Perri

**Affiliations:** 1 Department of Informatics, Modeling, Electronics and System Engineering, University of Calabria, 87036 Rende, Italy; cristian.sestito@unical.it (C.S.); f.spagnolo@dimes.unical.it (F.S.); 2 Department of Mechanical, Energy and Management Engineering, University of Calabria, 87036 Rende, Italy

**Keywords:** hardware accelerators, convolutional neural networks, transposed convolution, super resolution imaging, field programmable gate array (FPGA)

## Abstract

Nowadays, computer vision relies heavily on convolutional neural networks (CNNs) to perform complex and accurate tasks. Among them, super-resolution CNNs represent a meaningful example, due to the presence of both convolutional (CONV) and transposed convolutional (TCONV) layers. While the former exploit multiply-and-accumulate (MAC) operations to extract features of interest from incoming feature maps (*fmaps*), the latter perform MACs to tune the spatial resolution of the received *fmaps* properly. The ever-growing real-time and low-power requirements of modern computer vision applications represent a stimulus for the research community to investigate the deployment of CNNs on well-suited hardware platforms, such as field programmable gate arrays (FPGAs). FPGAs are widely recognized as valid candidates for trading off computational speed and power consumption, thanks to their flexibility and their capability to also deal with computationally intensive models. In order to reduce the number of operations to be performed, this paper presents a novel hardware-oriented algorithm able to efficiently accelerate both CONVs and TCONVs. The proposed strategy was validated by employing it within a reconfigurable hardware accelerator purposely designed to adapt itself to different operating modes set at run-time. When characterized using the Xilinx XC7K410T FPGA device, the proposed accelerator achieved a throughput of up to 2022.2 GOPS and, in comparison to state-of-the-art competitors, it reached an energy efficiency up to 2.3 times higher, without compromising the overall accuracy.

## 1. Introduction

In the last few years, deep learning algorithms, particularly convolutional neural networks (CNNs), have attracted considerable interest in several computer vision tasks, ranging from object detection [1] to image classification [2] and segmentation [3]. In such applications, the ever-growing success of CNNs is accompanied by a continuous increase in both accuracy and computational complexity. As an example, in the case of image classification, moving from the eight-layered AlexNet [4] to the 152-layered ResNet [5] the error rates have been reduced by more than 10%, but the amount of performed multiply-and-accumulate (MAC) operations has increased by more than 80%. Such a trend makes evident that ad-hoc designed hardware accelerators are essential for deploying CNN algorithms in real-time and power-constrained systems [6].

Most recently, the capability of reconstructing high-resolution images from low-resolution ones by means of pixel estimation, which is known as super resolution (SR) imaging, has become crucial in several applications, such as video surveillance, medical diagnosis, and remote sensing. Also in this field, CNNs have gained enormous popularity [7] and, thanks to the ability of learned filters to extrapolate new features from low-resolution images, they have demonstrated appreciable quality improvements with respect to conventional methods [8,9]. Unfortunately, because of the different nature of the final task to be accomplished, the existing hardware architectures designed to accelerate CNNs for object detection and classification are not well suited for SR imaging applications. Indeed, as a distinctive feature, in order to up-sample low-resolution images, CNN-based SR algorithms typically adopt transposed convolutional (TCONV) layers [10] that, with their computational complexity up to 6.75 times higher than traditional convolutional (CONV) layers, represent the most critical component of CNNs [11]. Moreover, in comparison to CONVs, TCONV layers require more complex strategies to access data memory, and make skipping operations necessary to manage the incoming pixels properly [12]. In order to overcome the aforementioned issues, several algorithms have been proposed [11,13,14,15] to transform TCONV into CONV layers by pre-processing either the input data or the filter coefficients. However, when implemented in hardware, these methods can show several drawbacks. Furthermore, most of the existing hardware designs are not configurable at run-time to support the different kernel sizes commonly demanded in CNNs for SR images [7,10]. Thereby, they use an ad-hoc tailored accelerator for each layer of the network, thus dramatically affecting the design effort, the energy efficiency, and the application flexibility.

To overcome the aforementioned issues, this paper presents a novel hardware-oriented algorithm that converts TCONV into CONV layers efficiently, without the requirement of any pre-processing. The main contributions of this work are summarized as follows:

- A comprehensive evaluation of the state-of-the-art TCONV algorithms suitable for implementation in hardware is provided.
- An original TCONV approach, thought to avoid complex remapping of filter coefficients and suitable for exploitation also in CONV operations, is presented.
- A flexible reconfigurable hardware accelerator is proposed. It was purposely designed to adapt itself at run-time to two operating modes and to different kernel sizes, as required to support all operations employed in both CONV and TCONV layers.
- For evaluation purposes, the novel method was exploited in the context of SR imaging, and the proposed reconfigurable hardware architecture was used to accelerate the popular fast super resolution CNN (FSRCNN) [10]. The experiments, performed on the Xilinx XC7K410T field programmable gate array (FPGA) chip, demonstrated the benefits of the proposed approach in terms of area occupancy and energy saving over several state-of-the-art counterparts. In fact, the new accelerator exhibited a logic resource requirement and a power consumption up to ~63% and ~48% lower, respectively, than previous designs [11,13,14,15,16,17]. The adopted parallelism and the achieved 227 MHz running frequency allow the above advantages to be obtained without compromising the competitiveness of the proposed design in terms of speed performance.

The reminder of this paper is structured as follows: Section 2 provides a background and a survey of previous works; the novel algorithm and the hardware architecture on-purpose designed are presented in Section 3 and Section 4; the experimental results are discussed in Section 5, which also includes a comparison to state-of-the-art accelerators implemented on the FPGA in terms of hardware characteristics and quality metrics. Finally, Section 6 concludes this manuscript.

## 2. Background and Related Works

The CNNs employed in SR imaging tasks [7] often include a feature extractor, consisting of several cascaded CONV layers, followed by an up-sampling stage consisting of a certain number of cascaded TCONV layers. The generic layer receives a volume of *M* input feature maps (*ifmaps*), each of a *H_i_* × *W_i_* size, and a set of *N* filters {*F*_0_, *F*_1_,…, *F*_*N*−1_}, each consisting of *M* kernels of a *k* × *k* size. The specific operations performed by the layer produce a volume of *N* output feature maps (*ofmaps*), each of a *H_o_* × *W_o_* size, with *H_o_* and *W_o_* being defined as $H_{o}=\left(S_{D}\times H_{i}+2P\right)-k+1$ and $W_{o}=\left(S_{D}\times W_{i}+2P\right)-k+1$, *S_D_* and *P* being, respectively, the up-sampling factor and the size of padding on the borders. 

**Figure 1:**
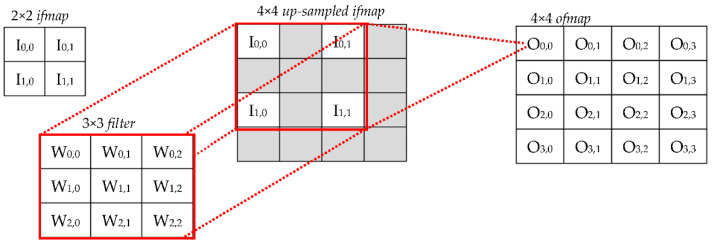
An example of the operations performed by a TCONV to process a 2 × 2 *ifmap* with a 3 × 3 filter when *S_D_* = 2.

In the case of CONVs, *S_D_* = 1 and, to generate the *h*-th *ofmap*, the volume of *ifmaps* is convolved with the corresponding filter *F_h_*. Then, the *M* results obtained in this way are summed up by a pixel-wise addition. Conversely, a TCONV layer refers to *S_D_* > 1 and requires the generic *ifmap* to be preliminarily up-sampled by interleaving actual input activations with *S_D_* − 1 additional rows and columns. After this, the operations involved are the same as those of a conventional CONV layer. The example illustrated in Figure 1 shows the operations performed to process a 2 × 2 *ifmap* with a 3 × 3 filter when *S_D_* = 2. It is worth noting that the additional elements introduced in the up-sampled *ifmap* can be filled either by zeros [18] (in the following, this approach is named the zero-TCONV) or by interpolating the nearest neighboring (NN) values to reduce possible chessboard effects [19]. Regardless, knowing the size *H_i_* × *W_i_* of the original *ifmap*, the up-sampling factor *S_D_*, and the size *P* of padding on the borders, the size *H_o_* × *W_o_* of the up-sampled *ifmap* is given by Equation (1).

Since they process up-sampled *ifmaps*, it is obvious that, with respect to CONVs, TCONVs require more MAC operations and larger amounts of data memory. Unfortunately, these characteristics may represent a bottleneck for those application scenarios in which real time and low power are mandatory. For this reason, designing ad-hoc hardware accelerators suitable for exploitation also within time- and power-constrained operating environments has recently received a great deal of attention [11,12,13,14,15,16,17,19,20,21,22,23]. Among the possible hardware realization platforms, FPGAs are widely recognized as powerful solutions [11,13,15,17,20] for merging the benefits from custom hardware designs, such as computational parallelism and limited energy consumption, with the strengths of software designs, including reconfigurability and short time to market.

While several of the existing hardware designs support both CONVs and TCONVs [11,13,14,15,16,17,19,21], some of them are tailored to accomplish only TCONVs [12,22,23]. As an example, the FPGA accelerator proposed in our previous work [12] deals with the input-oriented method (IOM) to reduce, or completely avoid, useless operations, corresponding to multiplications by zero, introduced by the conventional zero-TCONVs’ up-sampling approach. This is made possible by computing the products between each input pixel and the *k* × *k* elements of the filter, and then properly arranging the *k* × *k* results within the *ofmap*. Obviously, as a drawback, designs [12,22,23] need either additional buffers or auxiliary computing resources, or both, to manage row/column overlaps. Moreover, they may result quite inefficient when the CNN model being accelerated also uses CONV layers, as happens in the case of SR imaging applications [10,11].

The designs recently presented in [11,13,14,15] overcome the aforementioned issues by exploiting uniform accelerators for both CONVs and TCONVs. Starting from an analysis of the input-oriented method (IOM), and with the objective of avoiding overlapping on input activations, the computational scheme proposed in [11] performs an inverse mapping on the filter coefficients. More specifically, the transform deconvolution into convolution (TDC) approach [11] converts each filter of a TCONV into *S_D_*^2^ smaller sub-filters according to the relative position of the original input activations within the up-sampled *ifmap*. Due to this splitting strategy, several locations within the sub-filters contain zero values, thus causing unbalanced computations. Moreover, the configuration (i.e., size and number of sub-filters) depends on *SD*. Therefore, the splitting process has to be performed offline and the pre-processed filters must be stored on chip, thus limiting the possibility of reconfiguring at run-time the architecture to accelerate different CNNs.

As observed in [13], when the zero-TCONV approach is used, the filter coefficients that are being multiplied by zero activations can be removed by decomposing filters into several sub-blocks. Also for this decomposition algorithm, the filters must be pre-processed offline. Moreover, in order to remove unbalanced computations, an overall logic more complex than [11] is required.

To manage both TCONV and CONV operations, the hardware designs proposed in [14,15] decompose filters into smaller sub-blocks with different dimensions, according with the values of *k* and *SD*; then, to avoid filter reversal and zero padding on the borders, they apply a variant of the conventional Winograd algorithm. In such a case, unconventional computational modules, suitable for implementing operations involved in the Winograd transformation (such as inverse transformation of a matrix), are required.

The FlexiGAN architecture presented in [21] infers the conventional zero-TCONV operations, but, in order to improve the computational efficiency, it recognizes rows filled with zeros and skips them during the MAC operations. However, the auxiliary circuitry needed to properly reorganize the *ifmaps* and the filters significantly affect the logic and memory resource requirements, as well as the power consumption.

## 3. The Hardware-Oriented Algorithm Proposed to Convert TCONVs into CONVs

The novel algorithm here presented exploits a computational strategy quite different than previous works [11,12,13,14,15]. In contrast to [11,13,14,15], which manipulate the *k* × *k* filter coefficients to form smaller sub-blocks (thus introducing the necessity of offline elaborations), and with respect to [12] that re-arranges the position of output values within the *ofmaps* (leading to area and time overhead due to the management of the overlapping regions), it applies an unconventional remapping strategy directly to the incoming *ifmaps* values. From a hardware perspective, this means that: (1) The process occurs online and the preprocessing is not required, and (2) the result of the proposed algorithm can be outputted as soon as it is produced, thus avoiding additional time and buffering/computing resources. As a further advantage, the incoming *ifmaps* are not actually up-sampled, but instead are processed as if they were up-sampled with the zero-TCONV approach.

**Figure 2:**
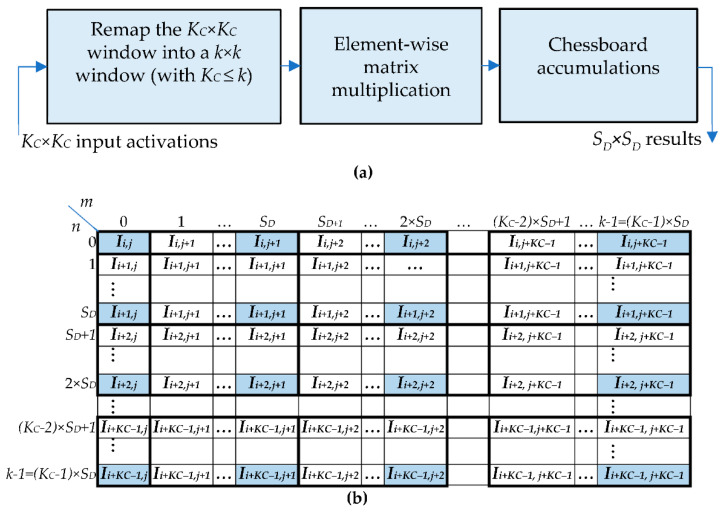
The novel algorithm: (**a**) The computational steps involved; (**b**) the remapping strategy.

In order to achieve high-speed performance and to prevent useless multiplications by zero, the proposed method was on-purpose made able to furnish *S_D_* × *S_D_* results in parallel for each computed *ofmap*. The steps illustrated in Figure 2a are performed to process the *K_C_ × K_C_* window of activations, with $K_{C}=\frac{k\;+\;S_{D}\;-\;1}{S_{D}}$. The generic sliding window received as input, with the first (i.e., the top-left) activation of the window being *I_i,j_* (with *i *= 0,…,*H_i_* − 1 and *j* = 0,…,*W_i_* − 1), is remapped within a *k* × *k* window; then, element-wise multiplications are performed between the remapped window and the *k* × *k* filter, followed by accumulations to produce *S_D_* × *S_D_* parallel results. The main innovation introduced with respect to the conventional approach and methods based on filter decomposition [11,13,14,15] is the remapping of the *K_C_ × K_C_* input activations within the sliding window *RI*. The latter is formed as illustrated in Figure 2b, which also shows the local row and column indices *m* and *n*, both varying from 0 to k−1. The remapped window is obtained by applying the following basic rules:

- The first activation *I_i,j_* is assigned to the local position (0,0) within the up-sampled window *RI* and replicated no more;
- The activations with a row index equal to *i* are replicated *S_D_* times horizontally;
- The activations with a column index equal to *j* are replicated *S_D_* times vertically;
- The activations with row and column indices varying, respectively, from *i* + 1 to *i* + *K_C_* − 2 and from *j* + 1 to *j* + *K_C_* − 2, are replicated *S_D_* times vertically and *S_D_* times horizontally, thus forming *S_D_* × *S_D_* sub-windows, as illustrated in Figure 2b;
- If $\left(k-1\right)\;mod\;S_{D}=0$, the activations with a row index equal to *K_C_* − 1 are replicated *S_D_* times horizontally (this is the case illustrated in Figure 2b); otherwise, they are replicated $\left(k-1\right)\;mod\;S_{D}$ times;
- If $\left(k-1\right)\;mod\;S_{D}=0$, the activations with a column index equal to *K_C_* − 1 are replicated *S_D_* times vertically (this is the case illustrated in Figure 2b); otherwise, they are replicated $\left(k-1\right)\;mod\;S_{D}$ times.

The elements of the remapped window, obtained as explained above, are multiplied by the homologous filter coefficients *W_m,n_* that do not require any type of rearrangement. Then, the computed *k* × *k* products *PP_m,n_* are properly accumulated to finally provide the *S_D_* × *S_D_* parallel results $O_{i\times S_{D}+p,j\times S_{D}+q}$, with *p* and *q* varying from 0 to *S_D_* − 1. To take into account the up-sampling factor *S_D_*, the generic result $O_{i\times S_{D}+p,j\times S_{D}+q}$ must be computed by accumulating *K_C_* × *K_C_* products *PP_mm,nn_* picked up starting from the location $mm=i\times S_{D}$, $nn=j\times S_{D}$ and going on as in a chessboard with horizontal and vertical jumps of *S_D_* positions (i.e., with stride *S_D_*). However, it is worth noting that some jumps lead to values of *mm* and/or *nn* exceeding *k*, thus indexing unavailable products. Actually, referring to the *ifmap* currently processed as if it were up-sampled with the zero-TCONV approach, it is easy to verify that these missing products correspond to multiplications by zero. Therefore, they do not contribute to the accumulate operations and can simply be ignored. As a consequence, the results computed with the proposed strategy have the same values provided by the conventional zero-TCONV approach [18]. However, the method proposed here completely avoids multiplications by zero and filter partitioning. The software model of the proposed method is reported in Appendix A.

It is important to highlight that the remapping strategy proposed here is a different point of view of the methods based on filters decomposition [11,13,14,15]. Indeed, while the latter re-arrange filter coefficients to perform proper element-wise multiplications, the former re-arrange input activations. However, as discussed in Section 5, the proposed strategy is more efficient from the hardware perspective, because it allows online computations and does not require complex architectures to manage the remapping.

**Figure 3:**
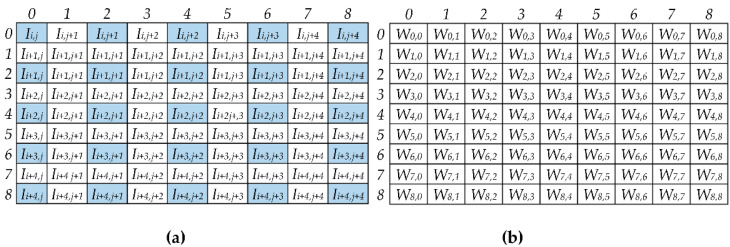
Example of computation with *k* = 9, *S_D_* = 2, and *K_C_* = 5: (**a**) The remapped window *RI*; (**b**) the filter *W*.

To better explain the novel computational scheme, let us consider the example in Figure 3 that refers to *k* = 9, *S_D_* = 2, and *K_C_* = 5. In this case, the local row and column indices *m* and *n* vary from 0 to 8. Therefore, for each input pixel *I_i,j_*, the above-explained basic rules lead to the remapped 9 × 9 window visible in Figure 3a, where the 5 × 5 elements of the original sliding window are highlighted in blue. It can be observed that the remapped window collects all of the data needed to compute the results $O_{i\times S_{D}+p,j\times S_{D}+q}$ contemporaneously, with indices *p* and *q*, used to locate the produced results within the *ofmap*, ranging between 0 and 1. Indeed, since *S_D_* = 2, the results $O_{i\times 2,\;j\times 2}$, $O_{i\times 2,\;j\times 2+1}$, $O_{i\times 2+1,\;j\times 2}$, and $O_{i\times 2+1,\;j\times 2+1}$ are computed as given in Equation (1).

(1) $$O_{i\times 2,\;j\times 2}=I_{i,j}\times W_{0,0}+I_{i,j+1}\;\times W_{0,2}+I_{i,j+2}\;\times W_{0,4}+I_{i,j+3}\;\times W_{0,6}+I_{i,j+4}\;\times W_{0,8}+\;+I_{i+1,j}\times W_{2,0}+I_{i+1,j+1}\times W_{2,2}+I_{i+1,j+2}\times W_{2,4}+I_{i+1,j+3}\times W_{2,6}+I_{i+1,j+4}\times W_{2,8}+\;+I_{i+2,j}\times W_{4,0}+I_{i+2,j+1}\times W_{4,2}+I_{i+2,j+2}\times W_{4,4}+I_{i+2,j+3}\times W_{4,6}+I_{i+2,j+4}\times W_{4,8}+\;+I_{i+3,j}\times W_{6,0}+I_{i+3,j+1}\times W_{6,2}+I_{i+3,j+2}\times W_{6,4}+I_{i+3,j+3}\times W_{6,6}+I_{i+3,j+4}\times W_{6,8}+\;+I_{i+4,j}\times W_{8,0}+I_{i+4,j+1}\times W_{8,2}+I_{i+4,j+2}\times W_{8,4}+I_{i+4,j+3}\times W_{8,6}+I_{i+4,j+4}\times W_{8,8}\;\;\;O_{i\times 2,\;j\times 2+1}=I_{i,j+1}\times W_{0,1}+I_{i,j+2}\;\times W_{0,3}+I_{i,j+3}\;\times W_{0,5}+I_{i,j+4}\times W_{0,7}+\;+I_{i+1,j+1}\times W_{2,1}+I_{i+1,j+2}\times W_{2,3}+I_{i+1,j+3}\times W_{2,5}+I_{i+1,j+4}\times W_{2,7}+\;+I_{i+2,j+1}\times W_{4,1}+I_{i+2,j+2}\times W_{4,3}+I_{i+2,j+3}\times W_{4,5}+I_{i+2,j+4}\times W_{4,7}+\;+I_{i+3,j+1}\times W_{6,1}+I_{i+3,j+2}\times W_{6,3}+I_{i+3,j+3}\times W_{6,5}+I_{i+3,j+4}\times W_{6,7}+\;+I_{i+4,j+1}\times W_{8,1}+I_{i+4,j+2}\times W_{8,3}+I_{i+4,j+3}\times W_{8,5}+I_{i+4,j+4}\times W_{8,7}\;\;\;O_{i\times 2+1,\;j\times 2}=I_{i+1,j}\times W_{1,0}+I_{i+1,j+1}\times W_{1,2}+I_{i+1,j+2}\times W_{1,4}+I_{i+1,j+3}\times W_{1,6}+I_{i+1,j+4}\times W_{1,8}+\;+I_{i+2,j}\times W_{3,0}+I_{i+2,j+1}\times W_{3,2}+I_{i+2,j+2}\times W_{3,4}+I_{i+2,j+3}\times W_{3,6}+I_{i+2,j+4}\times W_{3,8}+\;+I_{i+3,j}\times W_{5,0}+I_{i+3,j+1}\times W_{5,2}+I_{i+3,j+2}\times W_{5,4}+I_{i+3,j+3}\times W_{5,6}+I_{i+3,j+4}\times W_{5,8}+\;+I_{i+4,j}\times W_{7,0}+I_{i+4,j+1}\times W_{7,2}+I_{i+4,j+2}\times W_{7,4}+I_{i+4,j+3}\times W_{7,6}+I_{i+4,j+4}\times W_{7,8}\;\;\;O_{i\times 2+1,\;j\times 2+1}=I_{i+1,j+1}\times W_{1,1}+I_{i+1,j+2}\times W_{1,3}+I_{i+1,j+3}\times W_{1,5}+I_{i+1,j+4}\times W_{1,7}+\;+I_{i+2,j+1}\times W_{3,1}+I_{i+2,j+2}\times W_{3,3}+I_{i+2,j+3}\times W_{3,5}+I_{i+2j+,4}\times W_{3,7}+\;+I_{i+3,j+1}\times W_{5,1}+I_{i+3,j+2}\times W_{5,3}+I_{i+3,j+3}\times W_{5,5}+I_{i+3,j+4}\times W_{5,7}+\;+I_{i+4,j+1}\times W_{7,1}+I_{i+4,j+2}\times W_{7,3}+I_{i+4,j+3}\times W_{7,5}+I_{i+4,j+4}\times W_{7,7}$$

As expected, the results $O_{i\times 2+p,\;j\times 2+q}$, corresponding to *p* and/or *q* greater than zero, are obtained by accumulating less than *K_C_* × *K_C_* products, and the missing products are simply ignored, since they are related to multiplications by zero.

**Figure 4:**
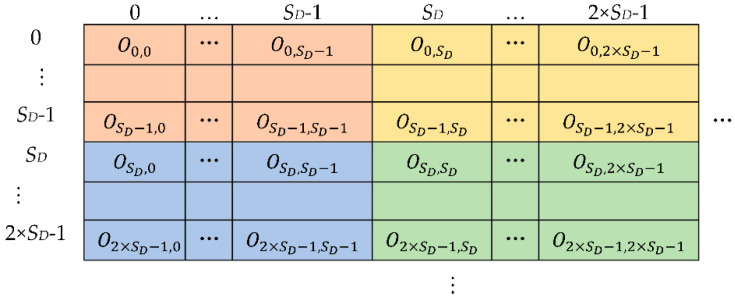
The arrangement of the computed results within the generic *ofmap*.

The computations described above are repeated for each pixel of the *ifmap* and, upon completion, the *H_i_* × *W_i_* groups of *S_D_* × *S_D_* results obtained in this way are arranged in the *ofmap*, as illustrated in Figure 4. In the figure, different colors are used to highlight each group of *S_D_* × *S_D_* results computed in parallel.

It is worth noting that when *S_D_* is 1, *K_C_* is equal to *k* and the sliding window does not require remapping operations; in such a case, the proposed algorithm performs a standard CONV. With the input volume consisting of *M ifmaps*, all of the computations described above must be repeated *M* times. The *M* intermediate *ofmaps* computed in this way are summed up to populate the volume of the expected *N ofmaps*.

## 4. The Proposed Run-Time Reconfigurable Hardware Accelerator

The novel method presented above to convert TCONVs into CONVs is employed within a reconfigurable hardware structure purposely designed to perform both CONVs and TCONVs by run-time, adapting itself to different operating modes. 

In order to achieve high computational speeds, the proposed hardware accelerator exploits a certain level of parallelism. In the following, it is shown that the *T_M_ ifmaps* and *T_N_* filters are processed at a time, with *T_M_* and *T_N_* varying at run-time in accordance with the current operation mode, the kernel size *k*, and the up-sampling factor *S_D_*. For the operations of the generic layer to be completed, regardless of whether it is a CONV or a TCONV layer, $\left\lceil \frac{M}{T_{M}}\rceil \times \lceil \frac{N}{T_{N}}\right\rceil$ steps are needed.

**Figure 5:**
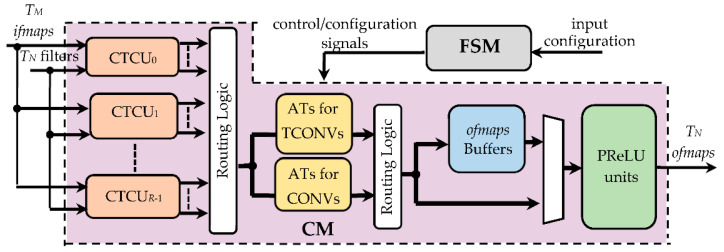
The top-level architecture of the proposed hardware accelerator.

Figure 5 depicts the top-level architecture of the proposed hardware accelerator that consists of a computational module (CM) and a finite state machine (FSM). The former receives, as inputs, *T_M_ ifmaps* and *T_N_* filters, each consisting of *T_M_* kernels collecting *k* × *k* coefficients, and provides *T_N_ ofmaps* at a time. Conversely, the FSM is fed with the input configuration, which sets the required operating mode (indicating whether CONVs or TCONVs must be performed), the kernel size *k*, the *fmap* sizes, and the window size *K_C_*, and furnishes proper control/configuration signals to the CM. Through these signals, the FSM configures the CM and supervises the overall data flow.

The CM splits the incoming *T_N_* filters into *R* groups and employs as many CONV/TCONV units (CTCUs). Each CTCU, depending on the received control and configuration signals, arranges data in proper sliding windows and executes either CONVs or TCONVs by processing the *T_M_ ifmaps* and its own $\left\lceil \frac{T_{N}}{R}\right\rceil$ filters. The results provided by the CTCUs are then dispatched to the subsequent modules passing through the routing logic purposely designed to take into account that the supported operating modes lead to different data flows. In fact, depending on whether CONVs or TCONVs are performed, the intermediate results related to the current *T_M_* input channels must be accumulated by the proper adder trees (ATs). Then, data must be routed either to the *ofmaps* buffers, which happens when the computation of the current *T_N_ ofmaps* is not yet completed, or, vice versa, to the parametric rectified linear units (PReLUs) that implement the linear rectification method demonstrated in [24].

**Figure 6:**
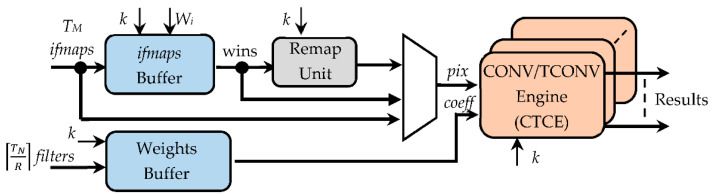
The architecture of the CONV/TCONV unit.

The generic CTCU is structured as illustrated in Figure 6. The *ifmaps* buffer (IFB) and the weights buffer (WB) collect, respectively, the *N_A_*-bit pixels of the incoming *T_M_ ifmaps* and the *N_W_*-bit coefficients of the received $\left\lceil \frac{T_{N}}{R}\right\rceil$ filters. In particular, the IFB circuit is responsible for arranging the *K_C_ × K_C_*-sized sliding windows that will be processed through the proposed algorithm. When TCONVs are executed, the remap unit (RU) performs the first step of the proposed approach. It implements the novel logic discussed above in Section 3 to remap the *T_M_ K_C_ × K_C_* sliding windows into as many *k* × *k* windows. The $\left\lceil \frac{T_{N}}{R}\right\rceil$ CONV/TCONV engines (CTCEs) execute the element-wise multiplications and the accumulations (steps 2 and 3 in Figure 2a); they receive the *T_M_* remapped windows and the filters coefficients as arranged, in the meantime, by the WB. When CONVs are executed with kernel sizes greater than 1, the RU is bypassed; thus, the IFB and WB feed directly the CTCE. In the case of 1 × 1 CONVs, both the IFB and the RU are bypassed, thus inputting the *ifmaps* directly to the CTCE.

**Figure 7:**
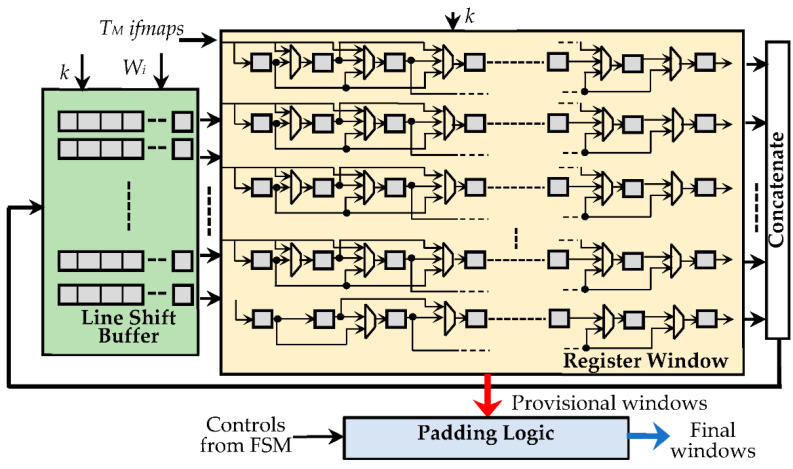
The organization of the *ifmaps* buffer.

While the WB uses just simple *N_W_*-bit shift registers, as shown in Figure 7, the IFB consists of three main parts:

- The register window (RW), composed of *K_M_* × *K_M_ N_A_*-bit registers, with *K_M_* being set to *T_M_* × *k* × *k*, thus ensuring that up to *T_M_ k* × *k* sliding windows can be accommodated at a time. The sparse multiplexing logic visible in Figure 7 guarantees that the used registers are properly cascaded according to the current value of *k*.
- The line shift buffer, used to locally store *W_i_* − *k* pixels of *k* − 1 rows of each received *ifmap*, and to perform shift operations, as conventionally required to properly accommodate the sliding windows during the overall computation.
- The padding logic, used to establish if the current sliding windows must be zero-padded, which occurs when the current anchor points are associated with the bordering pixels of the processed *ifmaps*.

**Figure 8:**
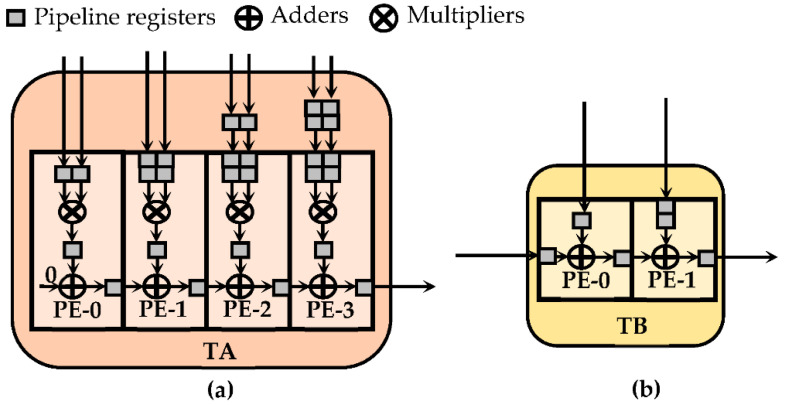
Example of PEs arranged in tiles: (**a**) TA with four Pes; (**b**) TB with two PEs.

**Figure 9:**
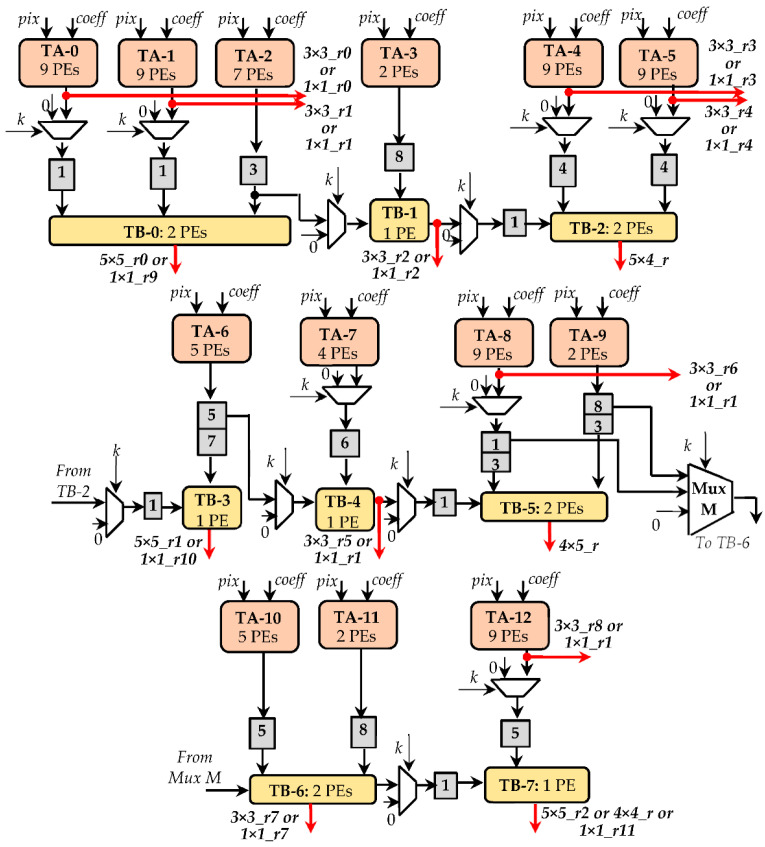
An example of the computations performed by CONV/TCONV engine (CTEC) when *k* is up to 9 and *S_D_* = 2.

Within the CTCE, multiplications and accumulation are performed, respectively, through two different pipeline sub-circuits, here named Type-A (TA) and Type-B (TB). As visible in Figure 8, each tile consists of several processing elements (PEs). The PEs inside the TAs execute MACs, whereas the PEs within the TBs perform two-operand additions. In order to provide a flexible architecture, suitable for performing both CONVs and TCONVs under different operating conditions, the CTCE exploits several TA and TB circuits, which are connected to one another by multiplexers. The latter allow to activate a specific path within the CTCE, depending on the currently processed kernel size. Taking into account that, as observed in the previous sections, at the parity of the kernel size, the TCONVs are more complex than CONVs, the employed sub-circuits TAs and TBs have been organized to comply with the computational capability required by TCONVs in the worst case, thus intrinsically being able to also satisfy the computational requirements of CONVs. As an example, Figure 9 illustrates the design of the CTCE when it has to comply with a 9 × 9 TCONVs at *S_D_* = 2. In this regard, 13 TAs and eight TBs are properly arranged to accomplish steps 2 and 3 of the proposed method. The TAs, consisting of 81 PEs, exploit as many multipliers to execute the element-wise matrix multiplication (step 2). Accumulators internal to the TAs, in conjunction with the 12 PEs provided by the TBs, perform the chessboard accumulations (step 3) to furnish the parallel results as in Equation (1). In Figure 9, the *S_D_* × *S_D_* parallel outputs are labeled as 5 × 5_r0, 5 × 4_r, 4 × 5_r, and 4 × 4_r, respectively. Subsequently, the external module ATs for TCONVs (visible in Figure 5) sums the referred outputs to the homologous results furnished by the other CTCEs operating in parallel. In addition, both TAs and TBs can be used to perform different CONVs, as follows:

1. Twelve 1 × 1 CONVs, whose results are 1 × 1_*ru*, with *u *= 0, …,11;
2. Nine 3 × 3 CONVs, with the furnished results being 3 × 3_*rx*, with *x* = 0,…,8;
3. Three 5 × 5 CONVs, whose results are 5 × 5_*ry*, with *y* = 0,…,2;
4. One 7 × 7 CONV; in this case the results 5 × 5_*r*0 and the 5 × 5_*r*1 are added by the external module ATs for CONVs;
5. One 9 × 9 CONV; in such a case the results 5 × 5_*r*0, the 5 × 4_*r*, 4 × 5_*r*, and 4 × 4_*r* are summed up by the external module ATs for CONVs.

Depending on which operation must be currently performed (e.g., CONVs or TCONVs) and based on the filter size *k*, the auxiliary multiplexing logic also depicted in Figure 9 coordinates the cooperation between TAs and TBs and guarantees that the different supported operations are performed correctly. The gray boxes represent the pipeline stages that, being deep as indicated by the reported numbers, time-align the performed computations.

It is worth noting that, in order to make the above-described CTCE able to support different up-sampling factors, just a few and simple modifications are required, either on the viable paths or on the compositions of the sub-circuits TAs and TBs. 

In order to explain the rest of the elaboration, let us refer to Figure 6 and suppose that the first computational step, related to the first *T_M_ ifmaps*, is just completed with the delivery of the first *T_N_* intermediate *ofmaps* as provided either by the module ATs for TCONVs or by the module ATs for CONVs. In the first step, such intermediate *ofmaps* are locally stored in the *ofmaps* buffer, waiting to be accumulated to the *T_N_* intermediate *ofmaps* that will be produced at the next step. The accumulation results are again locally stored in the buffer for the subsequent accumulations, and the operations go on in this way until the execution of the $\left\lceil \frac{M}{T_{M}}\right\rceil$-th step takes place, thus furnishing the final *T_N_ ofmaps*. Before being transferred to an external data memory, the latter are rectified by the PReLU units implementing the linear rectification approach demonstrated in [24]. 

All of the operations described above are executed $\left\lceil \frac{N}{T_{N}}\right\rceil$ times, i.e., until all the *N* final *ofmaps* are computed.

## 5. Experimental Results and Comparisons

As a case study, the real context of CNN-based SR imaging was referred to and the proposed approach was adopted to accelerate the popular FSRCNN model [10]. For this purpose, the hardware architecture described in the previous section was tailored to comply with the configurations summarized, layer by layer, in Table 1. Here, *M* and *N* refer to the number of *ifmaps* and *ofmaps*, *k* and *S_D_* are the kernel size and the up-sampling factor, and *T_M_* and *T_N_* are the number of *ifmaps* and *ofmaps* processed in parallel. It is worth noting that how many instances of the CTCU module are used, i.e., the value of *R*, is established at the design time to achieve a better trade-off between speed performances and area occupancy. For the referred case study, *R* = 12 was chosen, since it complies well with the requirements of the overall network model and allows reducing the inference time by more than 90% with respect to the case in which *R* = 1. Table 1 also reports the parameter *P_N_*, which indicates how many output values are computed in parallel for each of the *T_N_* furnished *ofmaps*. When the TCONV layer is executed, *P_N_* equals *S_D_* × *S_D_*, with *S_D_* being set to 2, 3, or 4, as established at the design time. The parameters *M*, *N*, *k*, and *S_D_* are elaborated by the FSM that: (1) The run-time configures the proposed hardware accelerator, thus ensuring that *T_M_* and *T_N_* change properly as required by each layer; (2) scans the various computational steps.

The novel accelerator exploits fixed-point arithmetic with activations and filters quantized, respectively, to 16 and 10 bits. Such a choice, which arises from a preliminary analysis conducted to evaluate the impact of different quantization levels on the quality of reconstructed images, allows improving the area occupancy by 60% and 18% with respect to 32- and 16-bit fixed-point versions, respectively, with detrimental effects on the quality of reconstructed images. Three different versions of the novel accelerator, each performing the TCONV layer with a specific up-sampling factor, have been designed by using the very high-speed integrated circuits hardware description language (VHDL) at the register transfer-level abstraction. Experimental tests were performed using the Xilinx ZCU102 development board [25], experiencing a frame rate of 192.3 fps when 256 × 256 input images were processed. Implementation results, obtained utilizing the Xilinx XC7K410T and XCZU9EG FPGA devices and the 2019.2 Vivado Design Suite, were collected in Table 2, reporting that:

- The amount of occupied look-up tables (LUTs), flip-flops (FFs), blocks of random access memory (BRAMs), and digital signal processing slices (DSPs);
- The power consumption, estimated through the switching activity values file (SAIF) that, referring to several benchmark images, taking into account the real activities of all nodes within the analyzed circuit;
- The speed performance, evaluated in terms of the maximum running frequency and the giga operations per second (GOPS), which is the ratio between the overall computational complexity of the referred model and the inference time;
- The energy efficiency (GOPS/W), which is defined as the ratio between the GOPS and the power consumption.

Table 2 also summarizes the implementation characteristics of representative state-of-the-art FPGA-based designs that, being devoted to the acceleration of CNNs for the SR imaging, have been selected as the direct competitors, even though they refer to somewhat different models from the original FSRCNN presented in [10]. As an example, while the designs proposed here were characterized referring to the whole model reported in Table 1, thus performing four cascaded CONV layers with *k* = 3 (i.e., layers 3, 4, 5, and 6), the accelerators presented in [11,15,17] refer to simplified models and perform only one CONV layer with *k *= 3. As a further simplification, to relieve the computational load, the design described in [17] replaces the TCONV with an efficient sub-pixel CONV (ESPCN) layer that provides up-sampled *ofmaps* through a periodic shuffling [26]. Conversely, the reconfigurable design presented in [13] refers to the original FSRCNN model, but it performs CONVs with kernels sizes ranging from 1 × 1 to 4 × 4 and changes the TCONV kernel size from 9 × 9 to 8 × 8.

In order to point out the main differences between the network models accelerated by the compared designs, they are referenced in Table 2 as FSRCNN(*x*,*y*,*z,w*). There, *x*, *y, z*, and *w* are, respectively, the number of *ofmaps* outputted by the first CONV layer, the number of *ofmaps* furnished by the subsequent CONV layers, the last excepted, the number of cascaded CONV layers with kernel size *k* = 3, and the TCONV kernel size.

By examining the results summarized in Table 2, it can be observed that, although referring to the most complex CNN model, due to their particularly efficient flexible architecture, the proposed accelerators lead to lower power consumptions. The power savings achieved with respect to [11,17] come from the capability of the proposed designs of the run-time adapting to different CONV kernel sizes. Without such a capability, the implementations characterized in [11,15,17] must employ a different ad-hoc architecture for each layer, thus negatively affecting the power consumption and the resources requirements.

In comparison to [11], the proposed XCK410T-based implementations save more than 53.7% LUTs, 22.3% FFs, and 14.3% DSPs, and improve the energy efficiency by up to 25.5%, which is also the result of avoiding multiplications with sparse filters, as required by [11]. These advantages are obtained even though the CNN model referenced in the novel designs is more complex than [11], which instead benefits from the reduced model complexity in terms of GOPS.

Table 2 clearly shows that the design demonstrated in [13] is particularly efficient in terms of occupied hardware resources. Nevertheless, when compared to [13], the novel accelerators implemented on the XCZU9EG chip consume ~3% less power and achieve up to ~16% higher GOPS, although they perform CONVs and TCONVs with greater kernel sizes and coefficients bit width.

The accelerator presented in [15] sacrifices a certain amount of hardware resources to implement a very deep pipeline, thus reaching the highest GOPS. However, such an advantage is obtained to the detriment of occupied LUTs, as a consequence of the Winograd algorithm implementation: In comparison to the proposed accelerator at a parity of implementation chip and *S_D_*, [15] performs ~9.5% more GOPS, but the amount of occupied LUTs is ~2.8 times higher.

Finally, from Table 2, it can be seen that, despite the simplifications introduced to reduce the computational complexity of the referred CNN model, at a parity of the up-sampling factor *S_D_* = 2, the design proposed in [17] occupies ~48.9% and ~77% more LUTs and DSPs than the novel accelerator targeting the XCK410T chip. Furthermore, the design presented here exhibits considerably improved speed performances and power consumption, which lead to a ~2.3 times higher energy efficiency.

For the sake of a fair analysis, the FSRCNN models referenced in Table were compared also in terms of the quality achieved at different up-scaling factors.

Software routines modeling the proposed accelerators were on-purpose written to process the popular *Set-5*, *Set-14*, and *B100* datasets and to evaluate the peak signal-to-noise ratio (PSNR) and the structural similarity (SSIM) [27]. Table 3 clearly shows that the strategy adopted here to transform TCONVs into CONVs does not affect the quality of reconstructed images. Indeed, in most of the analyzed cases, slightly improved PSNR and SSIM were achieved with respect to [11,13,17]. Furthermore, the small quality loss experienced in a few cases is well compensated by the benefits offered by the proposed method over its competitors in terms of some implementation characteristics. It is worth noting that the counterpart [15] was not included in the comparisons because the quality metrics furnished in the original paper are related to quite different datasets. 

**Figure 10:**
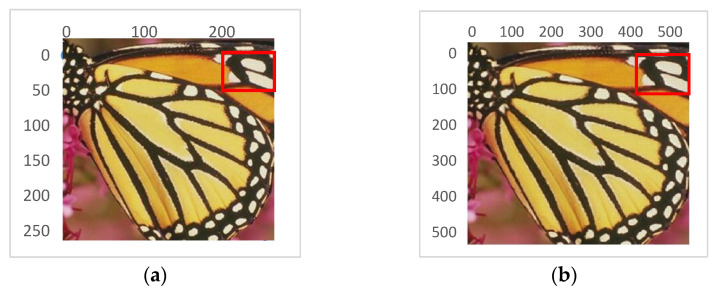
Sample results obtained with *S_D_* = 2: (**a**) The original image (from the public dataset *Set-5* http://mmlab.ie.cuhk.edu.hk/projects/FSRCNN.html (accessed on 25 August 2021) [28]); (**b**) the reconstructed image.

Finally, Figure 10 shows a sample image from the *Set-5* dataset that was up-sampled by using the proposed approach at *S_D_* = 2. As expected, the details were well reconstructed and, in this case, the achieved PSNR was 31.48 dB.

## 6. Conclusions

This paper presented an efficient hardware-oriented algorithm suitable to comply with the computational requirements of both the CONV and TCONV layers of many popular CNNs. The proposed approach was implemented by a flexible hardware architecture able for the run-time to adapt itself to different operating modes at various kernel and *fmap* sizes. In contrast to state-of-the-art counterparts, the novel strategy adopted here to transform TCONVs into CONVs does not require either pre-processing stages or offline kernels decompositions. Indeed, it exploits a simple reorganization of the sliding windows picked up from the incoming *ifmaps*. The capability of supporting different operating conditions and the simplicity of the remapping strategy led to reconfigurable hardware designs characterized by low power consumption, high-speed performance, and parsimonious utilization of logic resources.

In order to demonstrate the efficiency of the proposed approach, a fast super resolution CNN was referenced as a case study. Three versions of the novel reconfigurable hardware accelerator were implemented, each supporting a specific up-sampling factor. The characterization results obtained using the Xilinx XC7K410 FPGA device demonstrated that, although they refer to more complex CNN models, the proposed designs consume less power than their counterparts, occupying from 1.5 to 2.7 times less LUTs, and exhibiting an energy efficiency from 1.1 to 2.3 times higher. The tests performed on several datasets also demonstrated that the above advantages are achieved without compromising either the PSNR or the SSIM quality metrics.

## Figures and Tables

**Table 1:** The run-time configurations of the novel hardware accelerator related to the FSRCNN.

Layer	Op Mode	*M*	*N*	*k*	*S_D_*	*T_M_*	*T_N_*	*P_N_*
1	CONV	1	56	5	1	1	3 × *R*	1
2	CONV	56	12	1	1	56	*R*	1
3	CONV	12	12	3	1	9	*R*	1
4	CONV	12	12	3	1	9	*R*	1
5	CONV	12	12	3	1	9	*R*	1
6	CONV	12	12	3	1	9	*R*	1
7	CONV	12	56	1	1	12	3 × *R*	1
8	TCONV	56	1	9	2, 3, or 4	*R*	1	4, 9, or 16

**Table 2:** Implementation results and comparison with state-of-the-art competitors.

Accelerator		Proposed	Proposed	[11]	[13]	[15]	[17]
FPGA Device		XCK410T	XCZU9EG	XCK410T	XCVU095	XCZU9EG	XCVU9P
Model FSRCNN(*x*,*y*,*z*,*w*)		(56, 12, 4, 9)	(56, 12, 4, 9)	(25, 5, 1, 7)	(56, 12, 4, 8)	(32, 5, 1, 9)	(32, 5, 1, -) ^2^
Variable *k*		Yes, No	Yes, No	No, Yes	Yes, ^1^ Yes	No, No	No, No
Supported *S_D_*		2, 3, 4	2, 3, 4	2, 3, 4	2, 3, 4	2	2
#bits (activations, filters)		(16, 10)	(16, 10)	(13, 13)	(16, 8)	(16, 16)	(14, 10)
Max frequency [MHz]		227	250	130	200	200	200
LUTs	*S_D_* = 2	63.1 k	60.6 k	167 k	42 k	168.6 k	94 k
	*S_D_* = 3	56.9 k	54.6 k			-	-
	*S_D_* = 4	77.2 k	74.4 k			-	-
FFs	*S_D_* = 2	101.2 k	101.2 k	158 k	20 k	NA	19 k
	*S_D_* = 3	85.5 k	85.5 k			-	-
	*S_D_* = 4	122.8 k	122.8 k			-	-
BRAMs [Mb]	*S_D_* = 2	14.3	12	7.2	4.85	10.9	0.4
	*S_D_* = 3	14.3	12			-	-
	*S_D_* = 4	18.6	15.5			-	-
DSPs	*S_D_* = 2	1212	1212	1512	576	746	2146
	*S_D_* = 3	1140	1140			-	-
	*S_D_* = 4	1296	1296			-	-
Power [W]	*S_D_* = 2	3.6	3.8	5.4	3.71	NA	6.9
	*S_D_* = 3	3.5	3.85	-	-	-	-
	*S_D_* = 4	3.9	4	-	-	-	-
GOPS	*S_D_* = 2	654.3	720.6	780	605.6	795.2 ^3^	541.4 ^4^
	*S_D_* = 3	1223.5	1347.5	1576.3	1086.1	-	-
	*S_D_* = 4	2022.2	2227	2691	1868.8	-	-
GOPS/W	*S_D_* = 2	181.8	189.6	144.9	163.7	NA	78.5
	*S_D_* = 3	349.6	350	293	293.5	-	-
	*S_D_* = 4	518.5	556.8	500.2	505.1	-	-

^1^ The CONV kernel sizes range from 1 × 1 to 4 × 4. ^2^ The TCONV layer is replaced with an ESPCN layer. ^3^ Calculated considering the 120.4 frames per second declared in [15]. ^4^ Calculated considering the 60 frames per second declared in [17].

**Table 3:** Comparison results in terms of the PSNR and SSIM quality metrics.

		Proposed		[11]		[13]		[17]	
Dataset	*S_D_*	PSNR	SSIM	PSNR	SSIM	PSNR	SSIM	PSNR	SSIM
*Set-5*	2	35.68	0.9459	36.40	0.9527	35.85	NA	36.42	0.9529
*Set-14*	2	31.34	0.8650	32.21	0.9047	NA	NA	32.27	0.9045
*B100*	2	30.28	0.8765	31.15	0.8858	NA	NA	31.18	0.8859
*Set-5*	3	32.52	0.8816	32.48	0.9043	32.03	NA	NA	NA
*Set-14*	3	29.04	0.7975	29.03	0.8146	NA	NA	NA	NA
*B100*	3	28.27	0.7854	28.25	0.7808	NA	NA	NA	NA
*Set-5*	4	30.6	0.8577	30.17	0.8532	29.48	NA	NA	NA
*Set-14*	4	27.52	0.7480	27.24	0.7414	NA	NA	NA	NA
*B100*	4	26.90	0.7135	26.71	0.7041	NA	NA	NA	NA

**Figure A1:**
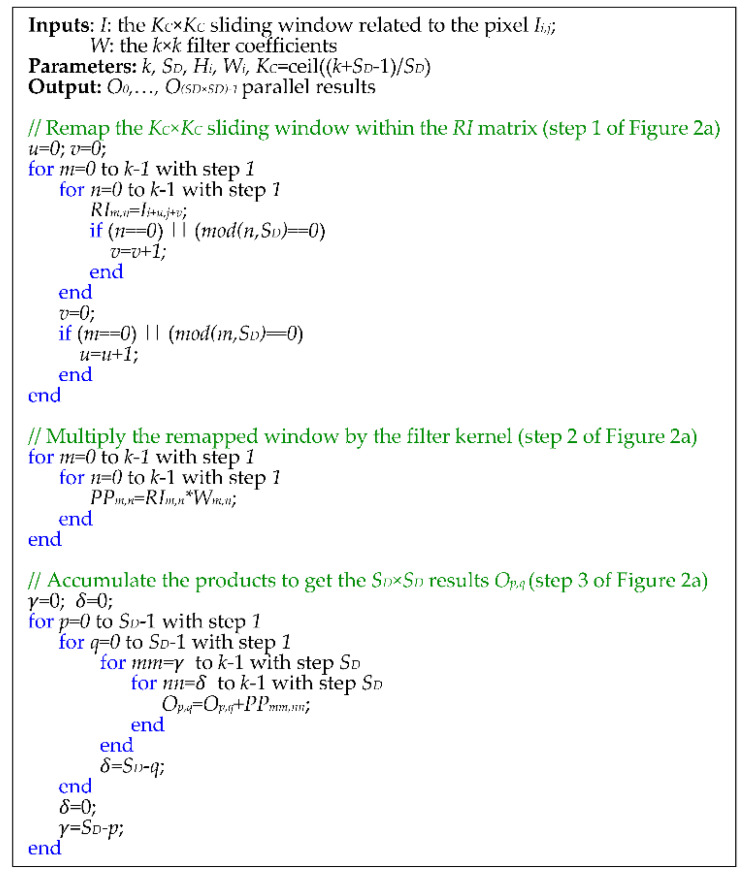
Software model of the proposed approach.

## Data Availability

Not applicable.

## Appendix A

Figure A1 details how the proposed approach processes the generic *K_C_ × K_C_* sliding window with the pixel *I_i_*_,*j*_ in the top-left position.
